# The cone dysfunction syndromes

**DOI:** 10.1136/bjophthalmol-2014-306505

**Published:** 2015-03-13

**Authors:** Jonathan Aboshiha, Adam M Dubis, Joseph Carroll, Alison J Hardcastle, Michel Michaelides

**Affiliations:** 1 UCL Institute of Ophthalmology, University College London, London, UK; 2 Moorfields Eye Hospital, London, UK; 3 Department of Ophthalmology, Medical College of Wisconsin, Milwaukee, Wisconsin, USA

**Keywords:** Retina, Genetics, Diagnostic tests/Investigation, Imaging, Vision

## Abstract

The cone dysfunction syndromes are a heterogeneous group of inherited, predominantly stationary retinal disorders characterised by reduced central vision and varying degrees of colour vision abnormalities, nystagmus and photophobia. This review details the following conditions: complete and incomplete achromatopsia, blue-cone monochromatism, oligocone trichromacy, bradyopsia and Bornholm eye disease. We describe the clinical, psychophysical, electrophysiological and imaging findings that are characteristic to each condition in order to aid their accurate diagnosis, as well as highlight some classically held notions about these diseases that have come to be challenged over the recent years. The latest data regarding the genetic aetiology and pathological changes observed in the cone dysfunction syndromes are discussed, and, where relevant, translational avenues of research, including completed and anticipated interventional clinical trials, for some of the diseases described herein will be presented. Finally, we briefly review the current management of these disorders.

## Introduction

The cone dysfunction syndromes (CDS) are a collection of heterogeneous inherited conditions, both in terms of their clinical characteristics and molecular genetic basis. They represent an important cause of lifelong visual impairment, with inherited retinal disorders being the second commonest cause of legal blindness in childhood and the leading cause among the working-age population in England and Wales.^1^ CDS have varying modes of genetic inheritance and have been classically described as stationary conditions in contrast to the progressive cone dystrophies.^2^ ^3^

Clinically, CDS are characterised by presentation at birth/early infancy with visual loss and variable degrees of colour vision abnormalities, nystagmus and photophobia, all of which reflect the dysfunction of the foveally concentrated cone cells that constitute approximately 5% of human photoreceptors. Given that these disease characteristics have an early onset and severely impair important behaviours of daily living such as facial recognition, reading and daylight vision, the consequent debilitating impact on patients’ lives is considerable.

In this review, we describe the phenotypic and genotypic features of CDS (excluding those solely of colour vision deficiency), including complete and incomplete achromatopsia (ACHM), blue-cone monochromatism (BCM), oligocone trichromacy (OT), bradyopsia and Bornholm eye disease (BED) (table 1). Given the new era of gene therapy interventions in human retinal disease,^4^ we will also briefly review the management and latest progress towards developing effective treatments.

**Table 1:** Summary of the clinical, genetic and electrophysiological characteristics of the cone dysfunction syndromes

Syndrome	Prevalence	Mode of inheritance	Typical BCVA (logMAR)	Typical refractive error	Nystagmus	Fundus findings	Colour vision	Typical ERG findings	Functional photoreceptors	Associated gene(s) (cytogenetic location)	Successful rescue of animal model/s
Complete achromatopsia *syn.* typical achromatopsia; rod monochromatism	1 in 30 000	Autosomal recessive	1.0	Often hypermetropic	Present	Usually normal	Absent	Absent cone responses; often normal rod responses	LW-cones: no MW-cones: no SW-cones: no Rods: yes	*CNGA3* (2q11.2) *CNGB3* (8q21-q22) *GNAT2* (1p13) *PDE6C* (10q24) *PDE6H* (12p13)	Yes
Incomplete achromatopsia *syn.* atypical achromatopsia	Uncertain	Autosomal recessive	0.6–1.0	Often hypermetropic	Present	Usually normal	Residual	Reduced or absent cone responses; often normal rod responses	LW-cones: possible MW-cones: possible SW-cones: possible Rods: yes	*CNGA3* (2q11.2) *CNGB3* (8q21-q22) *GNAT2* (1p13)	Yes
Blue-cone monochromatism *syn.* S-cone monochromatism; X-linked incomplete achromatopsia; X-linked atypical achromatopsia	1 in 100 000	X-linked recessive	0.6–1.0	Often myopic	Present	Usually myopic	Residual tritan discrimination	Reduced cone responses but with preserved S-cone responses; normal rod responses	LW-cones: no MW-cones: no SW-cones: yes Rods: yes	Principal opsin array mutational mechanisms on Xq28: (i) LCR deletion (approx. 40% cases) (ii) Non-homologous recombination between *OPN1LW*/*OPN1MW* resulting in a single gene in the array with a subsequent inactivating point mutation (approximately 60% of cases)	Yes
Oligocone trichromacy	Uncertain	Autosomal recessive	0.2–0.6	Equal prevalence of myopia and hypermetropia	Often absent	Normal	Normal	Reduced or absent cone responses; normal rod responses	LW-cones: yes MW-cones: yes SW-cones: yes Rods: yes	Possibly hypomorphic variants in the genes associated with achromatopsia	No
Bradyopsia *syn. RGS9*/*R9AP*-retinopathy	Rare	Autosomal recessive	0.2–0.6	Equal prevalence of myopia and hypermetropia	Often absent	Normal	Normal	Reduced/absent cone responses; the rod-specific ERG and the SBWF with ISI of 2 min are normal—however, the SBWF ERG with an ISCEV *standard* ISI of 20 s shows amplitude reduction, which is progressively less severe with increasing ISI, consistent with delayed recovery following the flash—thereby demonstrating the need for more extended testing than that mandated by ISCEV in the ERG Standard protocol	LW-cones: yes MW-cones: yes SW-cones: yes Rods: yes	*RGS9* (17q23-q24) *R9AP* (19q13.11)	No
Bornholm eye disease *syn.* X-linked cone dysfunction syndrome with dichromacy and myopia	Uncertain	X-linked recessive	0–0.8	Moderate to high myopia with astigmatism	Absent	Usually myopic	Deuteranopia or protanopia	Reduced cone responses; normal rod responses	LW-cones: yes, when observed with deuteranopia; no, when observed with protanopia MW-cones: yes, when observed with protanopia; no, when observed with deuteranopia SW-cones: yes Rods: yes	L/M interchange haplotypes (opsin array on Xq28)	No

BCVA, best-corrected visual acuity; ERG, electroretinography; ISCEV, International Society for Clinical Electrophysiology of Vision; ISI, inter-stimulus interval; LCR, locus control region; logMAR, logarithm of the minimum angle of resolution; LW, long wavelength; MW, middle wavelength; SBWF, single bright white flash; SW, short wavelength; *syn.*, synonym(s).

## Cone dysfunction syndromes

### Complete achromatopsia

Complete ACHM (*syn*. typical ACHM or rod monochromatism) is an autosomal-recessive condition associated with a lack of cone function,^5^ which affects about 1 in 30 000 people.^2^ It is characterised by presentation at birth/early infancy with pendular nystagmus, poor visual acuity (approximately logarithm of the minimum angle of resolution (logMAR) 1.0), a lack of colour vision and marked photophobia/hemeralopia. Patients may also demonstrate paradoxical pupillary constriction when transitioned from light to dark ambient conditions; the so-called Flynn phenomenon.^6^ Electroretinography (ERG) typically demonstrates absent cone responses and normal rod responses,^7^ ^8^ and psychophysical testing also reveals normal rod function but absent cone function.^9^ Hypermetropic refractive errors are common^10^ and fundus appearance is often normal, although macular changes can be observed that range from subtle retinal pigment epithelium (RPE) abnormalities to atrophy.

To date, five genes have been associated with ACHM, all encoding components of the cone-specific phototransduction cascade. Disease-causing sequence variants in these genes have been estimated to account for approximately 90% of ACHM cases.^11^ The first discovered, and most common, of these genes are *CNGA3*^12^ and *CNGB3*,^13^ which encode the α-subunits and β-subunits of the cGMP-gated cation channel, respectively. *CNGB3* mutations were first identified in a population of Micronesian islanders where the prevalence of complete ACHM was up to 3000 times that of other general populations; this was thought to be due to a typhoon that devastated the island in the 18th century,^14^ with all affected islanders able to trace their ancestry to a single typhoon survivor.^13^ Mutations in these two genes account for approximately 80% of all complete ACHM cases.^2^ ^15–17^

The most frequently identified mutation in *CNGB3* is the 1 base pair frameshift deletion c.1148delC (p.Thr383Ile fs*13), which accounts for >70% of *CNGB3* disease-causing alleles.^16–18^ There is far greater allelic heterogeneity in *CNGA3* disease-causing variants (over 80 described) compared with *CNGB3* (∼40). The majority of *CNGB3* variants identified to date are nonsense mutations, in direct contrast to the high proportion of missense mutations observed in *CNGA3*, suggesting that mutations that compromise the structural and functional integrity of the CNGA3 α-subunits are less well tolerated.

Disease-causing variants have been subsequently identified in (chronological order) *GNAT2*,^19^ which encodes the α-subunit of transducin (10 variants identified), *PDE6C,*^20^ encoding the α-subunit of cGMP phosphodiesterase (19 variants identified), and *PDE6H,*^21^ which encodes the inhibitory γ-subunit of the same enzyme (two variants identified). The genes *GNAT2*, *PDE6C* and *PDE6H* each comprise <2% of ACHM cases.^19^ ^21^ ^22^

In terms of functional and imaging assessment, there are no generalisable differences identified between the phenotype associated with the two most common complete ACHM genotypes (ie, *CNGA3* and *CNGB3*), although there is a marked degree of phenotypic variation observed within the genotypes.^18^ ^23^ ^24^ Spectral-domain optical coherence tomography (SD-OCT) imaging reveals a wide spectrum of photoreceptor integrity, ranging from a continuous inner segment ellipsoid (ISe) band at the fovea to outer retinal atrophy, and these findings have been both qualitatively and quantitatively assessed.^23–28^ Adaptive optics scanning light ophthalmoscopy (AOSLO) allows direct visualisation of individual human cone and rod photoreceptors in vivo*,*^29^ ^30^ and has identified residual cone structure in the majority of ACHM subjects imaged, although most of the cones have reduced reflectance and many ‘dark’ spaces are observed in the photoreceptor mosaic.^23^ ^31^ ^32^ More recently, split detection (non-confocal) imaging techniques have been coupled with existing AOSLO in order to visualise inner segment structure within the majority of the aforementioned ‘dark’ spaces seen on confocal AOSLO.^33^ These imaging results support the idea that cone structure in ACHM is disrupted, but not absent, and the degree of residual cone structure is highly variable between patients. These observations have significant implications for anticipated gene therapy clinical trial design in terms of patient selection and monitoring efficacy. Although no differences have been identified between *CNGA3* and *CNGB3* genotypes,^23^ ^24^ there is evidence that the *GNAT2* genotype may be associated with a greater degree of preservation of outer retinal architecture on SD-OCT and AOSLO assessment,^32^ and may retain residual cone function.^34^

ACHM in humans has been classically described as a non-progressive disease.^2^ ^7^ ^16^ ^35^ ^36^ Cross-sectional and longitudinal studies have found evidence of cone cell loss and/or progression over time,^27^ ^37–41^ although this is likely to occur very slowly, to a limited degree, and is also highly variable between patients with no definite age-dependency or genotype association.^23^ ^24^ ^41^

Rod photoreceptor function in ACHM has been classically described as normal,^7^ ^42^ although a number of studies have now reported abnormalities in rod-driven ERG responses^13^ ^23^ ^37^ ^43^ ^44^ and rod-derived dark-adaptation functions.^45^ ^46^ It is not yet clear whether a lack of functional cones might affect the rod photoreceptors themselves^47^ or the neural pathways that subserve them.^44^ ^48^ ^49^

Several studies have demonstrated the effectiveness of using gene-based or alternative therapeutic approaches to restore cone function in multiple animal models of ACHM of various genotypes.^50–54^ Given these promising results in animal models of the disease, there are plans to begin human gene replacement trials in the near future. One alternative therapeutic approach has been that of a recent phase I/II clinical study^55^ that delivered intravitreal ciliary neurotrophic factor to achromats with biallelic *CNGB3* variants; this failed to show any enhancement of cone function, although it has been suggested that the lack of assessment of residual cone number and placement during patient selection may have been a limiting factor in this study.^56^

### Incomplete achromatopsia

A small subset of patients with ACHM have an incomplete form of ACHM associated with residual colour vision as detected by psychophysical methods^57^ ^58^ and mildly better visual acuity (logMAR 0.6–1.0) than complete achromats.^2^ ^59^

The first genotype to be associated with incomplete ACHM was *CNGA3*.^15^ ^43^ It has been suggested that the *CNGA3* genotype might be unique in demonstrating residual cone function, given that most known *CNGB3* and *GNAT2* mutations (which constitute the two other most common ACHM genotypes by prevalence) result in premature termination and therefore in truncated and presumably non-functional proteins.^2^ However, both *GNAT2* and *CNGB3* patients have now been reported who appear to show residual cone function, as demonstrated by psychophysical tests, such as the Ishihara pseudoisochromatic colour plates and anomaloscope colour-matching tests, and/or residual cone ERG responses.^23^ ^34^ ^37^ ^60^ This finding in the latter genotype might not be entirely unexpected, given that *CNGA3* subunits alone have been shown to form functional homo-oligomeric channels in vitro.^61^

### Blue-cone monochromatism

This X-linked recessive condition is characterised by an absence of long (L)- (red) and middle (M)- (green) wavelength-sensitive cone function, the opsins for which are both encoded on the X-chromosome, while the short (S)- (blue) wavelength-sensitive opsin gene is located on chromosome 7.^62^ The prevalence is approximately 1 in 100 000, and affected males with BCM typically present at birth/early infancy with reduced visual acuity (logMAR 0.6–1.0), photophobia, nystagmus and are often myopic.^63^ Fundus examination reveals an otherwise normal myopic retina, but macular retinal pigment epithelial disturbance and atrophy have been noted in older patients.^64^ Vision in BCM is subserved by rod and S-cone photoreceptors alone, and consequently patients retain tritan discrimination,^63^ which has been reported to deteriorate with increasing illuminance.^65^ BCM can be clinically distinguished from ACHM by psychophysical and ERG assessment, with BCM demonstrating a profoundly reduced (but detectable) photopic ERG response and a preserved S-cone ERG,^66^ as well as by a corroborative family history, given the different modes of inheritance of the two conditions, and the often different refractive error. Nevertheless, the clinical distinction can be challenging in early infancy in a male patient and may not be definitively made until they are old enough to undertake detailed colour vision or ERG testing; the increased availability of genetic testing can now help to clarify the diagnosis.

The disease-causing variants in BCM fall into one of several categories, with the first two being the principal mechanisms: (i) a one-step pathway whereby the locus control region (LCR) is partially or completely deleted, thereby abolishing transcription of the opsin gene array^67^ (LCR is located upstream of the L-cone opsin (*OPN1LW*) and M-cone opsin (*OPN1MW*) genes, and controls transcription of the opsin array, resulting in only one opsin gene being expressed in any one photoreceptor^68^); (ii) a two-step mutation pathway, with the first step being non-homologous recombination between the L-opsin and M-opsin gene arrays resulting in a single-opsin gene in the array (often a hybrid gene), followed by a subsequent inactivating mutation (most commonly a missense variant) leading to a loss of functional L-cones and M-cones (the C203R missense mutation in a single L–M hybrid gene being the most frequently reported genotype^63^); (iii) the deletion of an entire exon in a single-opsin array gene;^63^ ^69^ or (iv) gene conversion transferring a mutation between *OPN1LW* and *OPN1MW.*^70^

SD-OCT analysis of patients with BCM has shown significant, although variable, macular thinning,^25^ ^71^ ^72^ with focal ISe disruption observed in an area corresponding to the normal S-cone-free zone.^71^ Despite having been traditionally described as a stationary condition, Cideciyan *et al*^72^ noted a trend towards increased thinning of the foveal outer nuclear layer in older patients with BCM, and other studies have also found evidence of progression in BCM.^63^ ^67^ ^73^ There is evidence that patients with LCR deletions are more likely to have a typical non-progressive BCM phenotype.^74^

Confocal AOSLO imaging has demonstrated a disrupted cone mosaic with a reduced number of cones at the fovea (both reflective S-cones and non-reflective L-cones and M-cones) to that of about 25% of normal in non-LCR-related BCM, with evidence of greater loss of cone cells in LCR deletion-related BCM.^71^ ^72^ In addition to the identification of residual cone structure, there is also potential for intervention in the future given the fact that gene replacement therapy in adult dichromatic monkeys lacking the L-opsin gene has been shown to produce trichromatic visual behaviour^75^ and has also demonstrated restoration of cone function in a rat model of BCM.^76^

### Oligocone trichromacy

OT is characterised by severe impairment of cone function on ERG assessment coupled with normal or near-normal colour discrimination. It was first described in 1973 by Van Lith,^77^ who reported a boy that, despite his poor vision and reduced photopic ERG responses, had nearly normal colour vision. This was hypothesised to be due to a low number of normal functioning cones (from the Greek *oligos* for ‘few’), which retained their normal distribution proportions between the three cone types, hence preserving trichromatic vision. It is believed to be an autosomal-recessive condition, wherein patients present in early childhood with mild photophobia, nystagmus which may or may not be present, reduced visual acuity (logMAR 0.3–0.6), normal fundi and normal rod responses on ERG.^78^ ^79^ Cone ERGs are markedly reduced, with ERG evidence in some cases of predominantly inner retinal dysfunction.^78^ Strikingly, however, despite these features of a CDS, colour vision is largely within normal limits, which may result in underascertainment of cases of OT. Using foveal densitometry measurements, Keunen *et al*^80^ argued that these patients possessed a reduced number of foveal cones that otherwise retained normal function. Goldmann visual fields are normal,^60^ ^79^ with reports of generalised retinal sensitivity reduction with Humphrey static visual field testing.^81^ Although believed to be predominantly stationary,^77^ there is some evidence that in some patients at least there may be progression.^78^

The underlying molecular genetic basis remains uncertain. OT and/or a ‘marked incomplete ACHM-like’ phenotype have been reported in association with ‘hypomorphic’ mutations in the ACHM genes *CNGA3*,^81^ ^82^ *CNGB3*,^79^ *PDE6C*^79^ and *GNAT2*.^60^ However, some of these cases arguably have features more in keeping with incomplete ACHM per se rather than OT. In addition, only single heterozygous missense variants have been identified in other subjects, thereby rendering their significance currently unclear. Nevertheless, OT is likely to be heterogeneous both genotypically and phenotypically,^78^ ^79^ in keeping with other CDS and inherited retinal disease as a whole. This heterogeneity has been further elucidated by Michaelides *et al*,^83^ who used adaptive optics (AO) and SD-OCT to assess the integrity of the cone photoreceptor mosaic and found that patients examined with a typical OT phenotype had a reduced number of functional cones at the fovea with no structure visible outside the central fovea, thereby confirming the original hypothesis of the underlying basis of OT; whereas patients with an OT-like phenotype had a normal cone mosaic in terms of cone density and distribution, thus suggesting that in these latter cases the cones present are dysfunctional. This study also identified that OT and bradyopsia (*RGS9*/*R9AP*-associated retinopathy) cannot be distinguished on the basis of clinical findings alone, with both being associated with normal colour vision.^83^ Extended ERG testing beyond International Society for Clinical Electrophysiology of Vision (ISCEV) standard testing is needed to identify the pathognomonic electrophysiological findings in bradyopsia (see the following section).^84–86^ There is evidence that these disorders can also be distinguished with high-resolution AO imaging, with patients harbouring *RGS9*/*R9AP* variants having an intact cone photoreceptor mosaic compared to patients with OT.^83^ ^87^

### Bradyopsia

This condition was first reported in 1991 in four Dutch patients, who demonstrated an abnormally long interval of suppression in their ERG amplitude responses to the second of a pair of bright stimuli flashes. This was postulated to be due to a deficit in the normally fast regeneration of the visual pathway signalling processes.^86^ The term bradyopsia (Greek for slow vision) was devised in 2004 to describe this stationary retinal phenotype, wherein affected patients had difficulty in adapting to sudden changes in cone-mediated luminance levels and difficulty in seeing moving objects.^84^ However, it is now clear that these symptoms can also be seen in many other disorders of cone function including OT.^83^ Onset is in early childhood and is associated with delayed dark and light adaptation, mild photophobia, moderately reduced visual acuity, normal colour vision and normal fundi.^85^ ^88^ ^89^ In patients with bradyopsia, the rod-specific ERG, the red flash ERG under dark adaptation (both an early cone and later rod system component) and the single bright white flash (SBWF) with inter-stimulus interval (ISI) of 2 min are all normal. The SBWF ERGs with an ISCEV standard ISI of 20 s show amplitude reduction, which is progressively less severe with increasing ISI, consistent with delayed recovery following the flash, demonstrating the need for an extended ISI to obtain full recovery of the ERG following the previous flash.^85^ A generalised reduction or absence of cone responses is observed (pattern ERG, 30 Hz flicker and photopic ERGs).^85^

A similar murine ERG phenotype was subsequently identified wherein the affected mice lacked the protein RGS9. This protein significantly accelerates the hydrolysis of the α-transducin bound guanosine triphosphate to guanosine diphosphate, thus deactivating the enzyme cGMP-phosphodiesterase and causing a rise in cGMP within the photoreceptor, consequently allowing the cGMP-gated cation channels to reopen.^90^ A further protein, R9AP, anchors RGS9 to photoreceptor outer segment disc membranes and enhances its activity by up to 70-fold.^91^ Thus, RGS9 and R9AP play critical roles in enabling the rapid recovery of the phototransduction cascade after light stimulation. Recessive mutations in the genes encoding these two proteins, namely *RGS9* and *R9AP*, have since been identified in humans.^84^ ^85^ ^88^ ^89^ To date, 1 missense^84^ and 1 nonsense mutation^85^ have been reported in *RGS9*, while 5 insertions/deletions have been reported in *R9AP*.^84^ ^85^ ^88^

Patients with either *RGS9*/*R9AP-*retinopathy or OT have very similar clinical phenotypes, characterised by stationary cone dysfunction, mild photophobia, normal colour vision and normal fundi. However, cellular imaging may be an effective way to distinguish between these conditions: AOSLO imaging of OT reveals a sparse mosaic of cones remaining at the fovea; in direct contrast, *RGS9*/*R9AP-*retinopathy patients have a normal cone photoreceptor mosaic.^83^ ^87^ This is in keeping with findings from dark-adapted flicker ERGs performed with a dim stimulus that show a normal response initially, which becomes undetectable after 10 s stimulation, in *RGS9*/*R9AP-*retinopathy patients,^85^ suggesting that cones are not only present (as demonstrated by AOSLO) but are capable of normal function and thus potentially amenable to rescue.

### Bornholm eye disease

BED was first described in a large family that originated from the Danish island of Bornholm.^92^ Affected members displayed X-linked recessive infantile myopia/astigmatism and impaired visual acuity, with signs of optic nerve head hypoplasia, retinal pigmentary changes, deuteranopia and reduced cone responses on ERG.^92^ ^93^ Since then, patients with protanopic BED have also been identified^94^ ^95^ and the disorder can now be described as an X-linked CDS associated with myopia and dichromacy.

The condition was mapped by linkage analysis to Xq28 in the original Danish family.^93^ Subsequent genetic interrogation has shown that rare haplotypes (‘L/M interchange haplotypes’) at polymorphic positions in exon 3 of the opsin genes, that result from intermixing between L- and M-opsin genes, are the principal underlying genetic basis of BED.^74^ ^96^ Some of these interchange haplotypes have been shown to result in aberrant splicing of the opsin genes and a variable degree of exon 3 skipping.^74^ ^97^

There is SD-OCT and AOSLO evidence that patients with BED demonstrate reduced retinal thickness and a significantly disrupted cone mosaic, although to a variable degree, and the suggestion has been made that the number of cones expressing the aberrant pigment (given that there can be more than a 30-fold range in the L:M cone ratio between individuals^98^) may determine whether the aforementioned polymorphic opsin variants lead to the generalised cone dysfunction observed in BED.^99^

## Management of CDS

Although there are now promising results for therapeutic intervention in other inherited retinal conditions in humans, and successful rescue of animal models in some CDS has been demonstrated, there are at present no proven treatments. Current management includes correct diagnosis, increasingly aided by molecular genetic testing, in order to offer accurate prognosis and genetic counselling, as well as refractive correction, low visual aids and educational support. Tinted lenses can help with disabling photophobia, improving both ocular comfort and quality of vision, for example, deep red tints in ACHM to reduce rod saturation, or magenta tints in BCM that would in addition preserve transmission of blue light.^100^ An accurate molecular genetic diagnosis is becoming increasingly possible and pertinent, given the likely significance this will have in directing future gene therapy. This, allied with research in other modes of intervention such as neuroprotection and pharmacological approaches, means that hopefully a new era is dawning where these hitherto untreatable conditions may be amenable to successful intervention.

## References

[1] Liew G, Michaelides M, Bunce C A comparison of the causes of blindness certifications in England and Wales in working age adults (16–64 years), 1999–2000 with 2009–2010. BMJ Open 2014;4:e004015 10.1136/bmjopen-2013-004015 PMC3927710 24525390

[2] Michaelides M, Hunt DM, Moore AT The cone dysfunction syndromes. Br J Ophthalmol 2004;88:291–7. 10.1136/bjo.2003.027102 14736794 PMC1771989

[3] Michaelides M, Hardcastle AJ, Hunt DM, et al Progressive cone and cone-rod dystrophies: phenotypes and underlying molecular genetic basis. Surv Ophthalmol 2006;51:232–58. 10.1016/j.survophthal.2006.02.007 16644365

[4] Ginn SL, Alexander IE, Edelstein ML, et al Gene therapy clinical trials worldwide to 2012–an update. J Gene Med 2013;15:65–77. 10.1002/jgm.2698 23355455

[5] Sharpe LT, Stockman A, Jagle H, et al Opsin genes, cone photopigments and colour blindness. In: Gegenfurtner KR, Sharpe LT Color vision: from genes to perception. 1st edn Cambridge: Cambridge University Press, 2001:48–52.

[6] Simon GB, Abraham F, Melamed S Pingelapese achromatopsia: correlation between paradoxical pupillary response and clinical features. Br J Ophthalmol 2004;88:223–5. 10.1136/bjo.2003.027284 14736779 PMC1771990

[7] Andréasson S, Tornqvist K Electroretinograms in patients with achromatopsia. Acta Ophthalmologica 1991;69:711–16. 10.1111/j.1755-3768.1991.tb02048.x 1789084

[8] Kohl S, Baumann B, Broghammer M, et al Mutations in the CNGB3 gene encoding the β-subunit of the cone photoreceptor cGMP-gated channel are responsible for achromatopsia (ACHM3) linked to chromosome 8q21. Hum Mol Genet 2000;9:2107–16. 10.1093/hmg/9.14.2107 10958649

[9] Hess R, Nordby K Spatial and temporal limits of vision in the achromat. J Physiol 1986;371:365–85. 10.1113/jphysiol.1986.sp015981 3486271 PMC1192730

[10] Simunovic M, Moore A The cone dystrophies. Eye 1998;12:553–65. 10.1038/eye.1998.145 9775217

[11] Roosing S, Thiadens AA, Hoyng CB, et al Causes and consequences of inherited cone disorders. Prog Retin Eye Res 2014;42:1–26. 10.1016/j.preteyeres.2014.05.001 24857951

[12] Wissinger B, Jägle H, Kohl S, et al Human rod monochromacy: linkage analysis and mapping of a cone photoreceptor expressed candidate gene on chromosome 2q11. Genomics 1998;51:325–31. 10.1006/geno.1998.5390 9721202

[13] Sundin OH, Yang J-M, Li Y, et al Genetic basis of total colourblindness among the Pingelapese islanders. Nat Genet 2000;25:289–93. 10.1038/77162 10888875

[14] Brody J, Hussels I, Brink E, et al Hereditary blindness among Pingelapese people of Eastern Caroline islands. Lancet 1970;295:1253–7. 10.1016/S0140-6736(70)91740-X 4192495

[15] Wissinger B, Gamer D, Jägle H, et al CNGA3 mutations in hereditary cone photoreceptor disorders. Am J Human Genet 2001;69:722–37. 10.1086/323613 11536077 PMC1226059

[16] Johnson S Achromatopsia caused by novel mutations in both CNGA3 and CNGB3. J Med Genet 2004;41:20e–20. 10.1136/jmg.2003.011437 PMC1735666 14757870

[17] Kohl S, Varsanyi B, Antunes GA, et al CNGB3 mutations account for 50% of all cases with autosomal recessive achromatopsia. Eur J Hum Genet 2005;13:302–8. 10.1038/sj.ejhg.5201269 15657609

[18] Thiadens AA, Slingerland NW, Roosing S, et al Genetic etiology and clinical consequences of complete and incomplete achromatopsia. Ophthalmology 2009;116:1984–9. e1 10.1016/j.ophtha.2009.03.053 19592100

[19] Kohl S, Baumann B, Rosenberg T, et al Mutations in the cone photoreceptor G-Protein α-Subunit Gene GNAT2 in patients with achromatopsia. Am J Human Genet 2002;71:422–5. 10.1086/341835 12077706 PMC379175

[20] Chang B, Grau T, Dangel S, et al A homologous genetic basis of the murine cpfl1 mutant and human achromatopsia linked to mutations in the PDE6C gene. Proc Natl Acad Sci 2009;106:19581–6. 10.1073/pnas.0907720106 19887631 PMC2780790

[21] Kohl S, Coppieters F, Meire F, et al A nonsense mutation in PDE6H causes autosomal-recessive incomplete achromatopsia. Am J Human Genet 2012;91:527–32. 10.1016/j.ajhg.2012.07.006 22901948 PMC3511981

[22] Grau T, Artemyev NO, Rosenberg T, et al Decreased catalytic activity and altered activation properties of PDE6C mutants associated with autosomal recessive achromatopsia. Hum Mol Genet 2011;20:719–30. 10.1093/hmg/ddq517 21127010 PMC3269206

[23] Genead MA, Fishman GA, Rha J, et al Photoreceptor structure and function in patients with congenital achromatopsia. Invest Ophthalmol Vis Sci 2011;52:7298–308. 10.1167/iovs.11-7762 21778272 PMC3183969

[24] Sundaram V, Wilde C, Aboshiha J, et al Retinal structure and function in achromatopsia: implications for gene therapy. Ophthalmology 2014;121:234–45. 10.1016/j.ophtha.2013.08.017 24148654 PMC3895408

[25] Barthelmes D, Sutter FK, Kurz-Levin MM, et al Quantitative analysis of OCT characteristics in patients with achromatopsia and blue-cone monochromatism. Invest Ophthalmol Vis Sci 2006;47:1161–6. 10.1167/iovs.05-0783 16505054

[26] Varsányi B, Somfai GM, Lesch B, et al Optical coherence tomography of the macula in congenital achromatopsia. Invest Ophthalmol Vis Sci 2007;48:2249–53. 10.1167/iovs.06-1173 17460287

[27] Thomas MG, Kumar A, Kohl S, et al High-resolution in vivo imaging in achromatopsia. Ophthalmology 2011;118:882–7. 10.1016/j.ophtha.2010.08.053 21211844

[28] Greenberg JP, Sherman J, Zweifel SA, et al Spectral-domain optical coherence tomography staging and autofluorescence imaging in achromatopsia. JAMA Ophthalmol 2014;132:437–45. 10.1001/jamaophthalmol.2013.7987 24504161 PMC4423754

[29] Roorda A, Williams DR The arrangement of the three cone classes in the living human eye. Nature 1999;397:520–2. 10.1038/17383 10028967

[30] Dubra A, Sulai Y, Norris JL, et al Noninvasive imaging of the human rod photoreceptor mosaic using a confocal adaptive optics scanning ophthalmoscope. Biomed Opt Express 2011;2:1864–76. 10.1364/BOE.2.001864 21750765 PMC3130574

[31] Carroll J, Choi SS, Williams DR In vivo imaging of the photoreceptor mosaic of a rod monochromat. Vision Res 2008;48:2564–8. 10.1016/j.visres.2008.04.006 18499214 PMC2582057

[32] Dubis AM, Cooper RF, Aboshiha J, et al Genotype-dependent variability in residual cone structure in achromatopsia: towards developing metrics for assessing cone health. Invest Ophthalmol Vis Sci 2014;55:7303–11. 10.1167/iovs.14-14225 25277229 PMC4235328

[33] Scoles D, Sulai YN, Langlo CS, et al In vivo imaging of human cone photoreceptor inner segments. Invest Ophthalmol Vis Sci 2014;55:4244–51. 10.1167/iovs.14-14542 24906859 PMC4095721

[34] Stockman A, Smithson HE, Michaelides M, et al Residual cone vision without α-transducin. J Vis 2007;7:8 10.1167/7.4.8 17461692

[35] Varsányi B, Wissinger B, Kohl S, et al Clinical and genetic features of Hungarian achromatopsia patients. Mol Vis 2005;11:996–1001. 16319819

[36] Thiadens AAHJ, den Hollander AI, Roosing S, et al Homozygosity mapping reveals PDE6C mutations in patients with early-onset cone photoreceptor disorders. Am J Human Genet 2009;85:240–7. 10.1016/j.ajhg.2009.06.016 19615668 PMC2725240

[37] Khan NW, Wissinger B, Kohl S, et al CNGB3 achromatopsia with progressive loss of residual cone function and impaired rod-mediated function. Invest Ophthalmol Vis Sci 2007;48:3864–71. 10.1167/iovs.06-1521 17652762

[38] Thiadens AA, Somervuo V, van den Born LI, et al Progressive loss of cones in achromatopsia: an imaging study using spectral-domain optical coherence tomography. Invest Ophthalmol Vis Sci 2010;51:5952–7. 10.1167/iovs.10-5680 20574029

[39] Thomas MG, McLean RJ, Kohl S, et al Early signs of longitudinal progressive cone photoreceptor degeneration in achromatopsia. Br J Ophthalmol 2012;96:1232–6. 10.1136/bjophthalmol-2012-301737 22790432

[40] Fahim AT, Khan NW, Zahid S, et al Diagnostic fundus autofluorescence patterns in achromatopsia. Am J Ophthalmol 2013. 10.1016/j.ajo.2013.06.033 23972307

[41] Aboshiha J, Dubis AM, Cowing J, et al A prospective longitudinal study of retinal structure and function in achromatopsia. Invest Ophthalmol Vis Sci 2014;55:5733–43. 10.1167/iovs.14-14937 25103266 PMC4161486

[42] Kelly JP, Crognale MA, Weiss AH ERGs, cone-isolating VEPs and analytical techniques in children with cone dysfunction syndromes*. Doc Ophthalmol 2003;106:289–304. 10.1023/A:1022909328103 12737507

[43] Nishiguchi KM, Sandberg MA, Gorji N, et al Cone cGMP-gated channel mutations and clinical findings in patients with achromatopsia, macular degeneration, and other hereditary cone diseases. Hum Mutat 2005;25:248–58. 10.1002/humu.20142 15712225

[44] Moskowitz A, Hansen RM, Akula JD, et al Rod and rod-driven function in achromatopsia and blue cone monochromatism. Invest Ophthalmol Vis Sci 2009;50:950–8. 10.1167/iovs.08-2544 18824728 PMC2631615

[45] Simunovic MP, Regan BC, Mollon J Is color vision deficiency an advantage under scotopic conditions? Invest Ophthalmol Vis Sci 2001;42:3357–64. 11726645

[46] Aboshiha J, Luong V, Cowing J, et al Dark-Adaptation Functions in Molecularly Confirmed Achromatopsia and the Implications for Assessment in Retinal Therapy Trials. Invest Ophthalmol Vis Sci 2014;55:6340–9. 10.1167/iovs.14-14910 25168900 PMC4193759

[47] Cho K-I, Haque M, Wang J, et al Distinct and atypical intrinsic and extrinsic cell death pathways between photoreceptor cell types upon specific ablation of Ranbp2 in cone photoreceptors. PLoS Genet 2013;9:e1003555 10.1371/journal.pgen.1003555 23818861 PMC3688534

[48] Haverkamp S, Michalakis S, Claes E, et al Synaptic plasticity in CNGA3−/− mice: cone bipolar cells react on the missing cone input and form ectopic synapses with rods. J Neurosci 2006;26:5248–55. 10.1523/JNEUROSCI.4483-05.2006 16687517 PMC6674253

[49] Baseler HA, Brewer AA, Sharpe LT, et al Reorganization of human cortical maps caused by inherited photoreceptor abnormalities. Nat Neurosci 2002;5:364–70. 10.1038/nn817 11914722

[50] Alexander JJ, Umino Y, Everhart D, et al Restoration of cone vision in a mouse model of achromatopsia. Nat Med 2007;13:685–7. 10.1038/nm1596 17515894 PMC3985124

[51] Komaromy AM, Alexander JJ, Rowlan JS, et al Gene therapy rescues cone function in congenital achromatopsia. Hum Mol Genet 2010;19:2581–93. 10.1093/hmg/ddq136 20378608 PMC2883338

[52] Michalakis S, Mühlfriedel R, Tanimoto N, et al Restoration of cone vision in the CNGA3−/− mouse model of congenital complete lack of cone photoreceptor function. Mol Ther 2010;18:2057–63. 10.1038/mt.2010.149 20628362 PMC2997579

[53] Carvalho LS, Xu J, Pearson RA, et al Long-term and age-dependent restoration of visual function in a mouse model of CNGB3-associated achromatopsia following gene therapy. Hum Mol Genet 2011;20:3161–75. 10.1093/hmg/ddr218 21576125 PMC3140821

[54] Wen R, Tao W, Li Y, et al CNTF and retina. Prog Retin Eye Res 2012;31:136–51. 10.1016/j.preteyeres.2011.11.005 22182585 PMC3288688

[55] Zein WM, Jeffrey BG, Wiley HE, et al CNGB3-achromatopsia clinical trial with CNTF: diminished rod pathway responses with no evidence of improvement in cone function. Invest Ophthalmol Vis Sci 2014;55:6301–8. 10.1167/iovs.14-14860 25205868 PMC4191169

[56] Langlo C, Dubis A, Michaelides M, Carroll J Comment: CNGB3-achromatopsia clinical trial with CNTF: diminished rod pathway responses with no evidence of improvement in cone function. Invest Ophthalmol Vis Sci 2014;56:1505. 10.1167/iovs.14-14860 PMC4191169 25205868

[57] Smith V, Pokorny J, Newell F Autosomal recessive incomplete achromatopsia with protan luminosity function. Ophthalmologica 1978;177:197–207. 10.1159/000308767 309579

[58] Smith V, Pokorny J, Newell F Autosomal recessive incomplete achromatopsia with deutan luminosity. Am J Ophthalmol 1979;87:393 10.1016/0002-9394(79)90083-7 312021

[59] Kohl S Achromatopsia– Rod monochromacy. In Traboulsi E, ed. Genetic Diseases of the Eye. Oxford University Press. Oxford. 2011:402 9.

[60] Rosenberg T, Baumann B, Kohl S, et al Variant phenotypes of incomplete achromatopsia in two cousins with GNAT2 gene mutations. Invest Ophthalmol Vis Sci 2004;45:4256–62. 10.1167/iovs.04-0317 15557429

[61] Finn J, Krautwurst D, Schroeder J, et al Functional co-assembly among subunits of cyclic-nucleotide-activated, nonselective cation channels, and across species from nematode to human. Biophys J 1998;74:1333–45. 10.1016/S0006-3495(98)77846-4 9512030 PMC1299480

[62] Nathans J, Piantanida TP, Eddy RL, et al Molecular genetics of inherited variation in human color vision. Science 1986;232:203–10. 10.1126/science.3485310 3485310

[63] Gardner JC, Michaelides M, Holder GE, et al Blue cone monochromacy: causative mutations and associated phenotypes. Mol Vis 2009;15:876. 19421413 PMC2676201

[64] Weleber RG Infantile and childhood retinal blindness: a molecular perspective (The Franceschetti Lecture). Ophthalmic Genet 2002;23:71–97. 10.1076/opge.23.2.71.2214 12187427

[65] Young R, Price J Wavelength discrimination deteriorates with illumination in blue cone monochromats. Invest Ophthalmol Vis Sci 1985;26:1543–9. 3877028

[66] Gouras P, MacKay C Electroretinographic responses of the short-wavelength-sensitive cones. Invest Ophthalmol Vis Sci 1990;31:1203–9. 2365554

[67] Nathans J, Davenport CM, Maumenee IH, et al Molecular genetics of human blue cone monochromacy. Science 1989;245:831–8. 10.1126/science.2788922 2788922

[68] Wang Y, Macke JP, Merbs SL, et al A locus control region adjacent to the human red and green visual pigment genes. Neuron 1992;9:429–40. 10.1016/0896-6273(92)90181-C 1524826

[69] Ladekjaer-Mikkelsen A-S, Rosenberg T, Jørgensen A A new mechanism in blue cone monochromatism. Hum Genet 1996;98:403–8. 10.1007/s004390050229 8792812

[70] Reyniers E, Van Thienen MN, Meire F, et al Gene conversion between red and defective green opsin gene in blue cone monochromacy. Genomics 1995;29:323–8. 10.1006/geno.1995.9998 8666378

[71] Carroll J, Dubra A, Gardner JC, et al The effect of cone opsin mutations on retinal structure and the integrity of the photoreceptor mosaic. Invest Ophthalmol Vis Sci 2012;53:8006–15. 10.1167/iovs.12-11087 23139274 PMC3816954

[72] Cideciyan AV, Hufnagel RB, Carroll J, et al Human cone visual pigment deletions spare sufficient photoreceptors to warrant gene therapy. Hum Gene Ther 2013;24:993–1006. 10.1089/hum.2013.153 24067079 PMC3868405

[73] Michaelides M, Johnson S, Simunovic M, et al Blue cone monochromatism: a phenotype and genotype assessment with evidence of progressive loss of cone function in older individuals. Eye 2005;19:2–10. 10.1038/sj.eye.6701391 15094734

[74] Gardner JC, Liew G, Quan YH, et al Three different cone opsin gene array mutational mechanisms with genotype-phenotype correlation and functional investigation of cone opsin variants. Hum Mutat 2014;35:1354–62. 25168334 10.1002/humu.22679 PMC4285181

[75] Mancuso K, Hauswirth WW, Li Q, et al Gene therapy for red–green colour blindness in adult primates. Nature 2009;461:784–7. 10.1038/nature08401 19759534 PMC2782927

[76] Zhang Z, Pang J, Xia F, et al AAV-mediated gene therapy restores cone function in a rat with an M-cone Opsin deficiency, a model for blue cone monochromacy. Invest Ophthalmol Vis Sci 2011;52:E-Abstract 1403.

[77] Van Lith G General cone dysfunction without achromatopsia. Xth ISCERG Symposium; 1973:175–80. 10.1007/978-94-010-2697-0_17

[78] Michaelides M, Holder G, Bradshaw K, et al Oligocone trichromacy: a rare and unusual cone dysfunction syndrome. Br J Ophthalmol 2004;88:497–500. 10.1136/bjo.2003.028142 15031164 PMC1772119

[79] Andersen MK, Christoffersen NL, Sander B, et al Oligocone trichromacy: clinical and molecular genetic investigations. Invest Ophthalmol Vis Sci 2010;51:89–95. 10.1167/iovs.09-3988 19797231

[80] Keunen J, De Brabandere S, Liem A Foveal densitometry and color matching in oligocone trichromacy. In: Drum B, ed. Colour Vision Deficiencies XII: Springer, Netherlands, 1995:203–10.

[81] Vincent A, Wright T, Billingsley G, et al Oligocone trichromacy is part of the spectrum of CNGA3-related cone system disorders. Ophthalmic Genet 2011;32:107–13. 10.3109/13816810.2010.544366 21268679

[82] Reuter P, Koeppen K, Ladewig T, et al Mutations in CNGA3 impair trafficking or function of cone cyclic nucleotide-gated channels, resulting in achromatopsia. Hum Mutat 2008;29:1228–36. 10.1002/humu.20790 18521937

[83] Michaelides M, Rha J, Dees EW, et al Integrity of the cone photoreceptor mosaic in oligocone trichromacy. Invest Ophthalmol Vis Sci 2011;52:4757–64. 10.1167/iovs.10-6659 21436275 PMC3175968

[84] Nishiguchi K, Sandberg M, Kooijman A, et al Defects in RGS9 or its anchor protein R9AP in patients with slow photoreceptor deactivation. Nature 2004;427:75–8. 10.1038/nature02170 14702087

[85] Michaelides M, Li Z, Rana NA, et al Novel mutations and electrophysiologic findings in RGS9- and R9AP-associated retinal dysfunction (Bradyopsia). Ophthalmology 2010;117:120–7. 10.1016/j.ophtha.2009.06.011 19818506

[86] Kooijman A, Houtman A, Damhof A, et al Prolonged electro-retinal response suppression (PERRS) in patients with stationary subnormal visual acuity and photophobia. Doc Ophthalmol 1991;78:245–54. 10.1007/BF00165687 1790747

[87] Liyanage SE, Cooper RF, Ba-Abbad R, et al Imaging Photoreceptor Structure in Subjects with R9AP- and RGS9-associated Retinal Dysfunction (Bradyopsia). Invest Ophthalmol Vis Sci 2014;55:E-Abstract 259 10.1167/iovs.13-13020

[88] Cheng JY, Luu CD, Yong VH, et al Bradyopsia in an Asian man. Arch Ophthalmol 2007;125:1138–40. 10.1001/archopht.125.8.1138 17698770

[89] Hartong DT, Pott J-WR, Kooijman AC Six patients with bradyopsia (slow vision): clinical features and course of the disease. Ophthalmology 2007;114:2323–31. 10.1016/j.ophtha.2007.04.057 17826834

[90] Cowan CW, Fariss RN, Sokal I, et al High expression levels in cones of RGS9, the predominant GTPase accelerating protein of rods. Proc Natl Acad Sci 1998;95:5351–6. 10.1073/pnas.95.9.5351 9560279 PMC20264

[91] Lishko PV, Martemyanov KA, Hopp JA, et al Specific binding of RGS9-Gβ5L to protein anchor in photoreceptor membranes greatly enhances its catalytic activity. J Biol Chem 2002;277:24376–81. 10.1074/jbc.M203237200 12006596

[92] Haim M, Fledelius H X-linked myopia in danish family. Acta Ophthalmol 1988;66:450–6. 10.1111/j.1755-3768.1988.tb04039.x 3264103

[93] Schwartz M, Haim M, Skarsholm D X-linked myopia: Bornholm Eye Disease. Clin Genet 1990;38:281–6. 10.1111/j.1399-0004.1990.tb03582.x 1980096

[94] Young TL, Deeb SS, Ronan SM, et al X-linked high myopia associated with cone dysfunction. Arch Ophthalmol 2004;122:897–908. 10.1001/archopht.122.6.897 15197065

[95] Michaelides M, Johnson S, Bradshaw K, et al X-linked cone dysfunction syndrome with myopia and protanopia. Ophthalmology 2005;112:1448–54. 10.1016/j.ophtha.2005.02.021 15953640

[96] McClements M, Davies WI, Michaelides M, et al Variations in opsin coding sequences cause X-linked cone dysfunction syndrome with myopia and dichromacy. Invest Ophthalmol Vis Sci 2013;54:1361–9. 10.1167/iovs.12-11156 23322568 PMC3597186

[97] Ueyama H, Muraki-Oda S, Yamade S, et al Unique haplotype in exon 3 of cone opsin mRNA affects splicing of its precursor, leading to congenital color vision defect. Biochem Biophys Res Commun 2012;424:152–7. 10.1016/j.bbrc.2012.06.094 22732407

[98] Carroll J, Neitz J, Neitz M Estimates of L:M cone ratio from ERG flicker photometry and genetics. J Vis 2002;2:531–42. 10.1167/2.8.1 12678637

[99] Neitz J, Wagner-Schuman M, Dubra A, et al Cone Mosaic Disruption Caused by L/M Opsin Mutations in Bornholm Eye Disease. Invest Ophthalmol Vis Sci 2011;52:E-Abstract 4896.

[100] Schornack MM, Brown WL, Siemsen DW The use of tinted contact lenses in the management of achromatopsia. Optometry 2007;78:17–22. 10.1016/j.optm.2006.07.012 17208670

